# Learning needs of nursing postgraduates in Southwest China based on Hutchinson’s learning needs theory: a qualitative research

**DOI:** 10.1186/s12909-023-04217-0

**Published:** 2023-04-07

**Authors:** Suofei Zhang, Huijuan Ma, Xiaoli Zhu, Aifang Niu, Yu Luo

**Affiliations:** School of Nursing, Third Military Medical University/Army Medical University, No.30 Gaotanyan Street, Shapingba District, Chongqing, 400038 P.R. China

**Keywords:** Nurse education, Nursing postgraduates, Learning needs, Qualitative research

## Abstract

**Background:**

The process of education is mutual. The learning needs of students need to be paid attention to and can affect the learning outcome. In order to make nursing postgraduates’ curriculum better, meet the learning needs of students, and help to achieve the learning objectives of students, this study based on Hutchinson’s learning needs theory, aims to collect the learning experience of nursing graduates, as well as the gap between learning needs and target needs, and explore the benefits and barriers of nursing graduates in the curriculum learning. It may provide beneficial reference for further optimizing teaching content and improving teaching methods.

**Methods:**

This study was designed as a qualitative research. Purposive sampling was used to recruit 17 nursing postgraduates in the only two universities in Chongqing, Southwest China, 2021. Semi-structured, in-depth individual interviews were conducted to explore how they subjectively experience the benefits and demands of the professional curriculum. Data was analyzed using Colaizzi’s seven-step analysis.

**Results:**

Three main themes “clear learning cognition and goals,” “positive learning attitude,” and “the gap between learning goals and actual needs” were identified from the original data. Respectively, Subthemes concerning the first theme included “improve scientific research capabilities, enlighten thinking and broaden your horizons, and learn new knowledge and new skills.” Subthemes of the second theme were “improve abilities in practice and actively seek diversification of course content and format.” Subthemes of the third theme included “the course has a certain depth and breadth, but the course study does not meet the needs of scientific research, the course contents are theoretical, not knowing how to use the research method in certain conditions.”

**Conclusions:**

The learning needs of nursing postgraduates in Southwest China could be divided into two parts: benefits and barriers, among which the benefits were participants had clear learning goals and positive learning attitudes. When curriculum could not meet their needs, they actively sought methods (e.g., networks or off-campus resources) to close the gap between those needs and their goals. Follow-up educators should focus on learning needs and build curricula by optimizing the contents and methods of existing teaching resources.

## Introduction

In conjunction with the promotion of informatization reform in the field of teaching [1], universities worldwide are continuously innovating and reforming their undergraduate and postgraduate courses [2, 3]. In the postgraduate training process, the construction of the curriculum system is a basic component of work. For students, course quality is directly related to the construction of knowledge structures, the shaping of innovation capabilities, and the improvement of overall quality. Given these factors, ensuring course quality is essential for nurturing and improving their professional knowledge and skills [4, 5]. As such, it is an irreplaceable key aspect of postgraduate education [6, 7].

The number of students enrolled in master’s degree nursing programs continues to increase annually. Due to the COVID-19 pandemic and relevant technological developments, nursing faculties have faced challenges in both academic and clinical settings, with effects on the teaching environment, surrounding conditions, and implementation pathways [8, 9]. Today, multiple resources are available for undergraduate and postgraduate courses worldwide. For example, Massive Online Open Courses (MOOCs) help individuals across the globe access excellent teaching resources. Against the background of informatization teaching, China also offers core courses for postgraduates, including research courses with different characteristics. However, in the context of increasingly rich, shared, and open-curriculum resources, there is still a lack of evidence on whether existing teaching resources can meet current learning needs [1].

In China, training objectives have now been clarified for master’s degree programs in nursing, with aims congruent with programs offered in advanced countries across Europe and America. While a consensus has therefore been reached, program planning remains unrefined. Postgraduate teaching contents [10] have led to course designs developing in different professional directions, but the setting is basically the same. Each major is not a prominent point of focus; rather, students are exposed to a wide range of learning contents without any specialization. Moreover, the professional characteristics required are not clearly delineated, and do not meet the goal of training clinical experts. Given this context, it is important to understand how postgraduate nursing students perceive their current master’s degree training, as their needs and demands are critical for improving the training model [11].

Hutchinson’s learning needs theory was developed by Hutchinson and Waters in 1987 [12, 13]. It relates to target needs and learning needs. Target needs refer to the knowledge and skills that learners should master for possible target situations. As such, this pertains to future learning demands, including necessities (target curriculum features), lacks (target curriculum features minus what the learner already knows), and wants (what the learner believes they want and need). Necessities and lacks are objective, and thus determined by persons other than the learner, while wants are subjective, and thus generated by the learner. Target needs can be determined through a combination of means, including questionnaires, interviews, observations, consultations, and text analyses [14]. Hutchinson and Waters also explained learning needs pertaining to classroom motivation and how individuals learn. Learning needs refer to the degree of knowledge and abilities that learners need to master in the learning process. These demands are also generated to meet objective goals, and thus constitute the learning demand in progress. In this context, the classroom learning environment must generate the motivation to learn.

Students’ learning is a subjective activity and everyone has their own learning purposes. A better grasp of the learning needs of the course can help us choose some better teaching contents and teaching methods, and better reform the teaching methods and the choice of teaching contents[15]. Postgraduates are more particular, not passive learners, but have a strong capacity for independent learning, exploring knowledge purposefully and actively. Postgraduates have autonomous learning motivation and independent learning needs and goals, and typical representatives of adult learning. Hutchinson’s learning needs theory that we have chosen include three layers, including target needs layer, the learning needs layer and the gap between the target needs and the learning needs, which fit the target participants and the purpose of this study. Therefore, Hutchinson’s learning needs theory was chosen as the theoretical basis for the study. Most of the previous studies on postgraduate nursing students have focused on competency-based research, but there is a lack of research on the subjective learning needs and objective goals for nursing postgraduates’ curriculum learning. Our study intends to collect the learning experience of nursing graduates, as well as the gap between learning needs and target needs, and explore the benefits and barriers of nursing graduates in the curriculum learning. It may provide beneficial reference for further optimizing teaching content and improving teaching methods.

## Methods

The protocol of this research was submitted to the Medical Ethics Committee of the Army Medical University in Chongqing, China. After examining the protocol in the formal meeting, the committee granted this research with ethical exemption due to interview surveys being conducted in this research as well as the proposed research involving no greater than minimal risk to participants. This study was performed in accordance with the Declaration of Helsinki. A qualitative design was adopted in this study, which allowed for an in-depth insight into the learning needs among nursing postgraduates and the qualitative study followed the Standards for Reporting Qualitative Research (SRQR). Prior to data collection, participants received an information letter about the study and a consent form. This study was carried out under the fully willingness of participants who signed a consent form before participating in this study.

### Study design

This study used a qualitative approach employing face to face semi-structured interviews with nursing postgraduates. A qualitative descriptive design study was conducted to explore the learning needs of nursing postgraduates. In the process of in-depth interview, nursing postgraduates can continuously insight into the research phenomenon through in-depth review and excavation of learning experience, increase students’ understanding and understanding of learning goals and learning needs, reflect on the gap between achieving goals and needs, and inspire strategies to narrow the gap.

### Participants

Purposive sampling was used to recruit 17 nursing master’s students from two universities in Chongqing, China. The inclusion criteria were: (1) 2018–2020 grade (including 2017 deferred) graduate nursing students who volunteered their participation; (2) experience in a professional nursing master’s course; (3) could think clearly and accurately use language to express their feelings; (4) agreed to be interviewed. The exclusion criteria were: (1) unable or unwilling to participate in the survey due to illness or personal reasons. In qualitative studies, the sample size is determined based on whether the required information can reach an adequate saturation level [16].

### Data collection

This qualitative study adopted phenomenological research methods. On the premise that all interviewees were aware of the study’s aims and procedures and provided their written consent, we collected data through face-to-face, semi-structured, in-depth interviews. We ensured that their confidentiality was maintained and that their privacy was protected. The interviews were completed by interviewers and recorders. Interview locations were selected according to each interviewee’s wishes and conducted in a quiet, independent room that was suitable as an interview environment. In each case, the entire process was synchronously recorded via voice recorder. During the interviews, we adjusted the method, sequence, and contents of questions according to the specific situation. We used the methods of explanation, clarification, and follow-up to avoid inducement and suggestion, paid attention to non-verbal information from interviewees, and recorded information promptly. Each interview lasted 30 to 40 min. Upon conclusion, we asked the interviewees for their opinions on the interview for follow-up improvements. We also designed a semi-structured interview guide based on a literature review, field visits, and our research purpose (Table 1).

**Table 1:** Interview guide

Semi-structured interview outline
1. Can you share your experience of the current nursing curriculum?
2. Can you share your experience of the teaching methods and methods currently used in nursing courses?
3. Can you share your experience of the school’s teaching resources? In addition to the existing courses, what other courses do you think need to be offered? How to obtain these resources?
4. What do you think is the gap between the nursing curriculum and your preset goals?
5. What is the gap between the knowledge and abilities acquired through current studies and your preset goals? On a scale of 10, indicate how the contributions of your tutor, yourself, and the course should be distributed.
6. What do you think are the ways to solve the above problems?

### Data analysis

The data collection and analysis were conducted concurrently. The consensus transcripts were further processed using Colaizzi’s method in the following steps [17]: (1) The answers of each interviewee were carefully read by at least two interviewers, SZ and HM, to obtain a general feeling; (2) Phrases or sentences directly pertained to the learning experiences or the subjective perception of the interviewees were extracted from each transcript; (3) Repeatedly appearing and significant statements were marked onto one of a few “coded statements”. For instance, the description “I have known the basic knowledge required in clinical work for several years, and I came to learn mainly because I want to know how to do research.” was marked to a coded statement “Improve scientific research capabilities”; (4) These steps were repeated for each description; (5) The coded statements were collected and organized into a few themes or sub-themes. For instance, coded statements “Improve scientific research capabilities” and “Enlighten thinking and broaden horizon” were grouped into a theme of “Clear learning cognition and goals”. This analysis was continued for all descriptions until no new theme or sub-theme emerged; (6) At this time, the data collected by the interviewers were reviewed by at least two professors of nursing to ensure the accuracy and completeness of the data and to ensure the quality of the data.7) For final validation, the interviewers returned to all participants and discussed the findings with them and made appropriate adjustments to maximally reflect the true experiences and perceptions of the participants.

### Rigour

Data trustworthiness should be achieved among qualitative research findings, and trustworthiness was ensured through three aspects: credibility, dependability, and transferability[18]. To achieve credibility, agreement was sought among research team when recruiting participants. Additionally, our research team is knowledgeable and experienced with qualitative research and data analysis. To ensure dependability and transferability, a transparent methodological process was followed, and a thorough description of quotes was integrated with the findings.

### Self-of-the-researcher

I am a researcher and teacher of the nursing with 8 years of teaching experience, undertaking nursing postgraduates’ courses in theory and progress in higher nursing education, educational assessment and nursing education management. Our research group is dedicated to nursing education research, especially focusing on digital education, education resource sharing, intelligent education and other research. Relevant research findings were presented twice at the recent ICN Congress.

## Results

Our interviewees included 4 males and 13 females, with an average age of 26.5 years. All were postgraduate nursing students. Their research interests included nursing education, humanistic nursing, geriatric nursing, and psychological nursing (Table 2).

**Table 2:** Participants demographic characteristics(N = 17)

Participants	Gender	Age	Grade	Learning seniority(years)	Professional direction
N1	M	22	2019	2	Nursing education
N2	F	27	2019	2	Elderly care
N3	F	33	2019	2	Mental/Psychological Care
N4	M	28	2019	2	Humanistic nursing
N5	F	33	2019	2	Elderly care
N6	F	31	2018	3	Integrated Chinese and Western Medicine Nursing
N7	F	32	2018	3	Clinical nursing
N8	F	25	2017	4	Psychological Care
N9	M	24	2018	3	Rehabilitation nursing
N10	F	36	2019	2	Elderly care
N11	F	26	2018	3	Chronic disease care
N12	F	33	2019	2	Elderly care
N13	F	27	2019	2	Critical care
N14	F	27	2018	2	Psychological Care
N15	F	27	2018	3	Psychological Care
N16	M	26	2019	2	Humanistic nursing
N17	F	28	2019	2	Elderly care

**Fig. 1:**
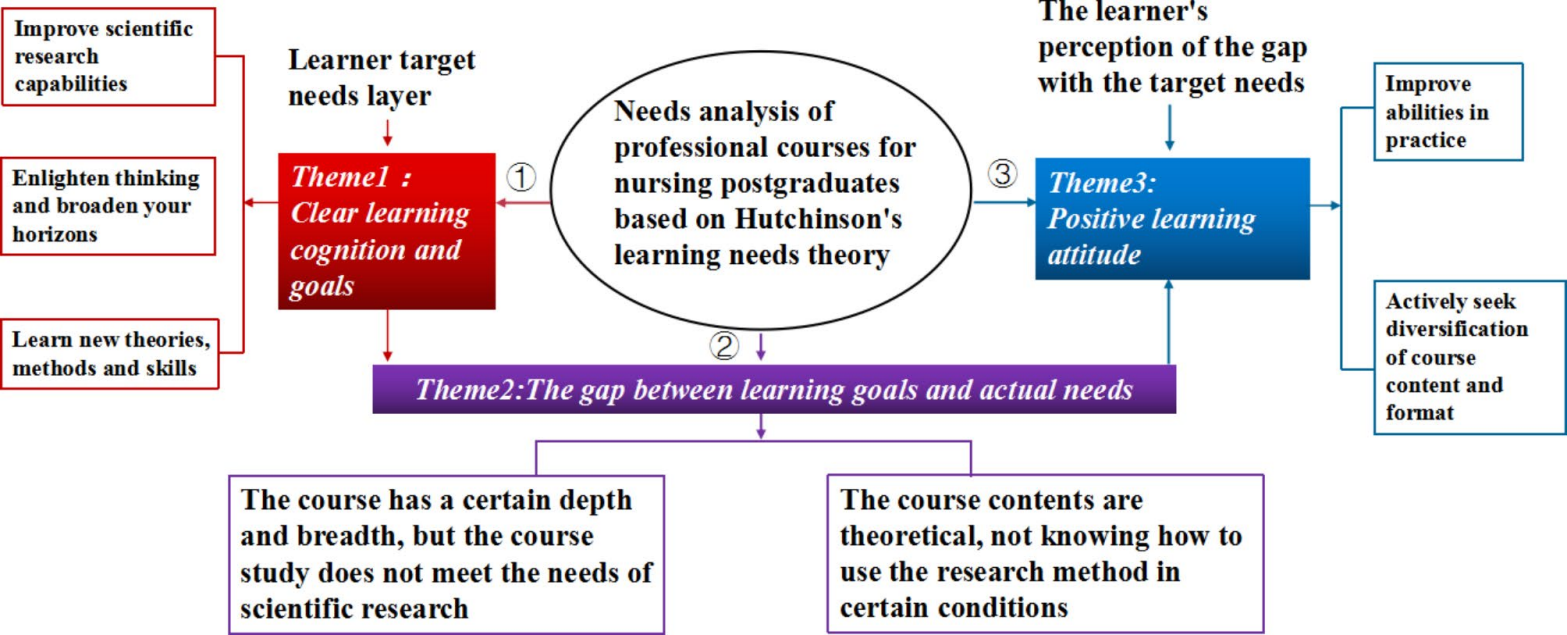
Three themes pertaining to the demand for professional courses among graduate nursing students

Based on our analysis, the demands for professional courses among nursing graduate students were categorized into three themes, namely, clear learning cognition and goals, positive learning attitude, and the gap between learning goals and real needs (Fig. 1).

### Theme 1: clear learning cognition and goals

The formation of the first theme mainly comes from the participants’ subjective experience of the course learning of nursing postgraduate’s curriculum. They can clearly express their self-learning needs during the interview process, including their hope to improve their scientific research ability, enlighten their thinking and broaden their horizons through the curriculum learning, and also hope to learn new knowledge and new technology through the curriculum. From the above aspects, we can see that nursing postgraduates have their clear learning cognition and goals for curriculum learning.

The first subtheme identified in relation to this theme was “improve scientific research capabilities.” Most interviewees said that the basic goal of a master’s degree course in nursing was to improve scientific research abilities through professional courses. Specific abilities included discovering and solving problems, using scientific research methods, and applying statistical methods. Relevant quotes were as follows:

*“From the perspective of a graduate student, the results will not be used to measure the quality of the course learning, but the scientific research results projected to the research stage… Whether it is to improve my ability for special research or to improve my ability through courses, the ultimate goal is still that I want to learn through a certain course to improve my corresponding ability and academic level.” (S01)*.

The second subtheme identified was “enlighten thinking and broaden your horizons.” These achievements were anticipated through course learning. While postgraduate students are expected to have higher knowledge than undergraduate students, the interviewees still desired exposure to new knowledge, technologies, and methods to stimulate their curiosity and promote new ideas. As some of them stated:

*“Our school’s resources are already very rich, but the school has created a platform for us to communicate with other schools. In the process of learning together, being exposed to outstanding talents from other universities has a significant effect. This kind of excellence puts people under pressure. To be precise, it’s the pressure that brings motivation, and my vision opens up at once.” (S07)*.

The third subtheme identified was “learn new theories, methods, and skills.” Some interviewees hoped to learn new theories, new methods, and new technologies related to their profession through systematic course learning. This is the first step toward good practice. As some of them noted:

*“The nursing theory course has cutting-edge content. When the teacher talks about it, she gives some examples. Anyone studying this theory will gain a deeper understanding of the theory and will speak with greater knowledge. This class can provide that opportunity; if you are interested in new insights, you can explore more deeply.” (S10)*.

### Theme 2: the gap between learning goals and actual needs

Our second theme is the gap between learning needs and actual needs. In the background, we mentioned that the learning demand is a demand in the future, while the actual demand is a demand in the process. The previous theme mentioned that students have a clear learning purpose, mainly to improve their scientific research ability. However, after learning at the current stage of the course, students show a certain sense of lack in the sense of learning convenience, which is mainly reflected in the fact that although the course has a certain depth and breadth, it can not fully meet the subsequent scientific research needs. The other outstanding performance is that the content of course learning is theoretical, teaching and practice are disconnected, and the theory can not be used in specific situations when applied.

The first subtheme identified was “the course has a certain depth and breadth, but the course study does not meet the needs of scientific research.” Most interviewees said that strong benefits were derived through professional graduate nursing courses. There are obvious differences in postgraduate courses where further exploration and expansion are offered. Some respondents observed:

*“When I was studying nursing ethics during my undergraduate study, I felt that the content was very empty, because I didn’t have the intention of doing scientific research in the undergraduate course. The purpose of the graduate study was very clear, and the course was more in-depth and thorough.” (S01)*.

*“Compared with the previous undergraduate courses, I think the graduate courses are richer. Pure theoretical teaching in the undergraduate setting is more limited. The graduate courses not only require you to understand what is going on but also know how to apply the knowledge.” (S03)*.

The second subtheme identified was “the course contents are theoretical, without providing guidance on how to use the research method in certain conditions.” For postgraduate education, some interviewees believed that three basic components were primarily emphasized (i.e., basic knowledge, technology, and skills). Thus, course learning did not improve the scientific research capabilities needed at the graduate level. In terms of acquiring advanced pioneering knowledge, the classroom setting provided far less information than lectures and academic conferences. Most students with master’s degrees in nursing had come from the clinical setting. Some relevant quotes were as follows:

*“There is still a gap between the course and my expectations. My direction is health promotion. I want to know more methods for health promotion, but this class did not provide more methods; while the course provided useful foundational knowledge with some benefits, my ability for further scientific research remains limited.” (S12)*.

### Theme 3: positive learning attitude

The third theme to emerge from our data analysis was positive learning attitude. Nursing graduate students are in the adult learning stage, and they have clear goals and autonomy. When the actual learning cannot meet the learning needs, they will find strategies to reduce the gap between learning needs and actual needs. Their positive learning attitude is mainly reflected in their ability to improve in the follow-up project practice. At the same time, in the background of the shared global education resources, they will actively seek more diversified learning methods and teaching resources.

**Fig. 2:**
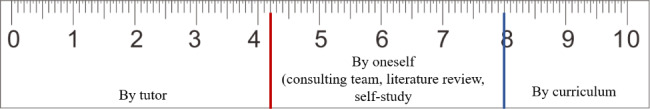
Ruler measuring 10 cm to evaluate three factors that affect abilities to be improved during the graduate stage

The first subtheme identified was “improve abilities in practice.” Because graduate students are in the adult learning stage and have clear learning goals, most interviewees used self-help to solve difficulties. They identified and addressed gaps during research projects, and gradually improved various abilities. This study also employed a subjective evaluation method wherein a 10 cm ruler was used to evaluate three factors that influenced ability improvements during the graduate stage, namely, the tutors, oneself (gaining information by oneself, self-study, seeking help), and the curriculum (Fig. 2). 17 participants conducted the ruler measure and the results were as follows:

Some student observations were as follows:

*“It’s certainly impossible to cover everything in terms of curriculum design, and to develop all one’s abilities. Later, some abilities are improved in the process of doing research in one’s own research direction.” (S05)*.

The second subtheme identified was “actively seek diversification of course contents and format.” While some interviewees said that course hours were insufficient and the contents were difficult to process, they gained knowledge and information through other courses, both online and within different disciplines. Some explained what this meant as follows:

*“Nursing and psychology actually overlap. I attended the psychology assessment and statistics stages of a course offered by the Department of Psychology, because nursing and psychology are both soft sciences, and the methods are the same. Such courses can be set as electives or other options can be taken. That course is divided into two stages: measurement and statistics. My research involves questionnaire compilation, so I was particularly concerned about those aspects.” (S02)*.

Student S12 expressed the subjective feelings about participating in online and offline courses in the process of actively seeking curriculum resources. In the process of seeking diversified teaching methods, innovative advice has been proposed to carry out nursing practice across schools and hospitals.

*“The shared courses of other schools are carried out in the form of online courses, but I still like offline courses, which have a stronger sense of benefit. In addition, if I can go to other school-affiliated hospitals for traineeship, it would be great. Look at the nursing styles of other hospitals. Hospitals are definitely different.” (S12)*.

## Discussion

Everything has two sides. In addition to paying attention to the students’ sense of autonomy about the course learning experience, we also need to pay attention to the course itself and possible problems in the teaching process. Based on these student experiences with nursing postgraduates’ curriculum, this study identified three benefits and two barriers .

### Benefits

The first benefit was clear cognition and clear learning goals. For both professional and academic graduate students in nursing, concepts and distinctions could be less clear across degree types, but the goals of course participants were highly similar. Most postgraduates hoped to improve their scientific research abilities. Postgraduate studies constitute an adult learning stage that assumes strong purposes and pertinence [19]. At this time, the most important ability that needs improvement is the ability to undertake scientific research; students have high course expectations and hope to take their first steps in this area. Indeed, most students entering postgraduate courses expect considerable benefits from research methodology courses [20–22], including nursing research, qualitative research, statistical methods, and advanced nursing theory/practice. Of these, nursing research methods and qualitative research were two courses offered at different schools. Nursing research methods also included qualitative research contents. Most participants also claimed to gain considerable benefit from taking a separate qualitative research course as they were able to conduct independent research after completing the course. This finding has important implications for the development and selection of relevant curricula and teaching contents. Guided by training plans and syllabuses, course developers should select and expand teaching contents that help students in a targeted and reasonable manner, thus improving their scientific research abilities as much as possible.

The second benefit was that course study could enlighten the mind and broaden horizons at the postgraduate stage. Skill development occurred not only in relation to evidence assessment but also concerning how to undertake research systematically. At this stage, cultivating the ability to identify and solve problems is a key secondary goal. Meanwhile, postgraduate study is a process in which students deepen their understanding of nursing. For example, the participants said that they had broadened their horizons and had their minds opened by participating in lectures concerning the frontiers of their discipline, and even learned about new areas. Through participation in presentations, some participants also said that they had been able to come to know experts in the field, thus establishing good teacher-student relationships. During the learning process, some participants explained that they had been able to contact other students through tutors and gain many new insights, which further enlightened their thinking and expanded their horizons [23, 24]. In this regard, curriculum learning not only facilitates actual ability improvements but also provides various other benefits. Here, teaching was found to be focused on making more opportunities available for students, including those pertaining to the learning environment, learning resources, and teaching methods.

The third benefit was a positive learning attitude and ability to seek learning resources and methods, both inside and outside school. Based on the results obtained using the 10 cm ruler, students who encountered difficulties with course learning mainly asked their tutors for help (42%) or relied on themselves (38%) (e.g., asking classmates, seeking resources through the platform, and self-study), whereas fewer received help through the curriculum (20%). These results highlight the importance of encouraging self-learning abilities among postgraduates, which promotes the learning effect. Compared with the undergraduate study stage, the postgraduate study stage is both more purposeful and likely to require stronger autonomous learning abilities. Most students who take professional courses have predetermined learning goals. When course studies do not meet these goals, students tend to solve problems and close this gap themselves; for example, the interviewees said that they would ask tutors and study teams for advice, conduct online searches, review the literature, or take online courses.

### Barriers

The first barrier was that the curriculum did not fully meet their scientific research needs. Although the course had a certain depth and breadth, most interviewees were still confused about scientific research after completing their studies. Nursing graduates start the graduate study stage with clear learning objectives. The first contact is the graduate professional course, and then enter the project research stage. However, after the course study, especially after the study of the methodology and statistics courses related to the follow-up research, it is found that the scientific research methods in the course are not enough to solve the problems encountered in the project research. Although the teaching content of postgraduate courses is set at a higher level of academic expectations, the teaching of new theories, new technologies and new knowledge is insufficient to meet the learning needs of postgraduate students. They pay more attention to scientific research methods, including scientific research design, scientific research methods, thesis writing methods, etc. Here comes the first barrier in curriculum learning. The students’ learning needs cannot be met, reflecting the problems that may exist in the education and curriculum content, and there is room for improvement. Some healthcare policymakers pointed out the current education does not entirely fulfill the goal of cultivating high-level practical talents[15] Therefore, we educators should pay more attention to students’ learning needs and learning objectives. Although students can narrow the gap by seeking some ways independently, educators should be more active in providing support for their needs and making beneficial attempts to help students achieve learning objectives.

Moreover, the knowledge acquired through course learning was insufficient for solving problems during scientific research and in the clinical setting. There comes the second barrier which refers to theoretical knowledge in the course is divorced from the practical application. At present, most of the classroom teaching of nursing research is still conducted in the form of theoretical teaching, discussion, classroom training, etc. The transformation of theoretical knowledge to practical application is still lacking, and the lack of ability to transfer and apply theoretical knowledge has led to a certain gap between students’ learning needs and objectives. The above problems suggest that we need to improve the course content and teaching methods. X. Shi et al. [15] demonstrated that there were a wide gap between school education and clinical work. Similarly, previous studies found room for improvement in clinical practice training in Master of Nursing science program [25, 26]. Advices should be taken into consideration that it is essential to optimize the clinical practice pattern to improve professional capacities[27]. Some educators undertook learning situation analyses, set curriculum contents according to actual student needs, and also use cases, practical teaching, and other methods in the classroom to strengthen the transfer and application of knowledge [28]. Researcher introduced the construction of a practice teaching system in MNS programs, such as strengthening the specialty construction, enhancing institutional collaboration and constructing a scientific evaluation system[29].

## Limitations

This study had the following limitations. First, approximately 70% of the participants were from the same university, which reduced the diversity of the sample. However, the researchers sought to collect relevant data through information saturation, which has been shown to be effective in previous research. Second, some interviews were conducted after the course had concluded, which may have resulted in recall bias. To ensure accuracy, the researchers prompted the interviewees concerned to list as many examples as possible and repeatedly confirmed them.

## Conclusions

Education programs should meet the internal (students, graduates and faculty) and external (employers and stakeholders) demands and be based on the national circumstances [30]. In Southwest China, postgraduate master’s nursing degree education is now undergoing reform, especially in relation to curriculum education. This study used Hutchinson’s learning needs theory to analyze the experiences and desires of graduate nursing students who were attending universities in this area. In summary, the participants had clear learning goals and positive learning attitudes during the curricular learning stage. When curriculum learning did not meet their needs, they actively sought methods to narrow the gap between relevant needs and goals (e.g., seeking networks or off-campus resources). More learning platforms and opportunities development are essential [31]. Follow-up educators should pay attention to current learning needs and build the curriculum by optimizing the contents and methods found in existing teaching resources. As both “shared education” and “inter-school education” will continue to feature in the foreseeable future, educators should focus on reorganizing and sharing teaching methods and resources, make fuller use of currently available excellent teaching resources, pay more attention to student needs and their sense of benefit, and provide students with professional graduate courses that meet their learning expectations.

## Data Availability

The datasets used and/or analyzed during the current study are available from the corresponding author upon reasonable request.
